# Prognostic and Predictive Biomarkers in Adult and Pediatric Gliomas: Toward Personalized Treatment

**DOI:** 10.3389/fonc.2014.00047

**Published:** 2014-03-24

**Authors:** Harry R. Haynes, Sandra Camelo-Piragua, Kathreena M. Kurian

**Affiliations:** ^1^Department of Neuropathology, Frenchay Hospital, Bristol, UK; ^2^Department of Neuropathology, University of Michigan, Ann Arbor, MI, USA

**Keywords:** gliomas, prognostic biomarkers, predictive biomarkers

## Abstract

It is increasingly clear that both adult and pediatric glial tumor entities represent collections of neoplastic lesions, each with individual pathological molecular events and treatment responses. In this review, we discuss the current prognostic biomarkers validated for clinical use or with future clinical validity for gliomas. Accurate prognostication is crucial for managing patients as treatments may be associated with high morbidity and the benefits of high risk interventions must be judged by the treating clinicians. We also review biomarkers with predictive validity, which may become clinically relevant with the development of targeted therapies for adult and pediatric gliomas.

## Introduction

At present, histological grading represents the most reliable, accepted overall indicator for the clinical outcome in adult and pediatric glioma patients (1). Gliomas are histologically classified according to the World Health Organization (WHO) classification system, which assigns a malignancy Grade I–IV (2). Traditionally, the higher the grade of gliomas, the worse the prognosis.

Primary glioblastoma (previously referred to as glioblastoma multiforme or GBM) develop *de novo*. This contrasts with secondary GBM, which undergo progressive malignant transformation from low grade diffuse astrocytoma (WHO Grade II) or anaplastic astrocytoma (WHO Grade III) (3). It has been estimated that 5% of all GBMs are secondary lesions (4). Secondary GBM patients are approximately 15 years younger than those with primary GBM and have a significantly better clinical outcome (5). Although, primary GBM cannot reliably be distinguished histologically from secondary GBM (6), their genetic profiles differ substantially as discussed below. This raises the possibility that they develop from differing neural precursor cells (7).

It is increasingly clear that GBM can be subdivided into tumor groups, which have fundamentally differing molecular drivers and varying treatment responses (8). The Cancer Genome Atlas (TCGA) project aims to establish the genetic and expression profiles for a wide range of tumors (9). Based on this and other profiling, GBM has been divided into four putative subtypes: proneural, neural, classical, and mesenchymal each with a unique biological behavior (10). However, Philips et al. identified alternative prognostic subclasses of high-grade astrocytomas: proneural, proliferative, and mesenchymal (11). The proneural subclass was enriched for neuronal lineage markers, occurred in younger patients and has a better prognosis. The proliferative and mesenchymal markers expressed neural stem cell markers and have worse clinical outcomes. Recurrent gliomas have been observed to shift expression patterns toward the mesenchymal subclass (12). The Philips proneural subtype resembles subsets from the TCGA proneural and neural subtypes, the Philips proliferative group resembles subsets from the TCGA proneural and classical. The mesenchymal subtypes overlap in both studies (13, 14).

The most recent data from the TCGA network has combined copy-number, DNA methylation, protein, mRNA, and microRNA (miRNA) expression profiles of 543 GBMs plus exome DNA sequencing in 291 glioblastomas (15). The key findings of this data will be discussed throughout this review.

It is essential that new tools are developed that better delineate the biological variants of gliomas. Without this more finessed approach, treatment targets may be missed and patients given toxic therapies not sufficiently targeted to their glioma subtype. Such a biomarker approach means that patients with the same histological diagnosis, tumor location, and co-morbidities may receive differing therapy based on the molecular characteristics of their tumors (16).

A prognostic biomarker is a tumor-specific trait that predicts clinical outcome regardless of treatment given. Conversely, a predictive biomarker predicts clinical response to a specific treatment or drug class (17). Using such biomarkers will allow personalized prognostic estimates to be given to patients with a subsequent customized treatment regime, maximizing effectiveness, and minimizing toxicity. Prognostic biomarkers can also be used for patient stratification in clinical trial design, to ensure balance in the arms of randomized control trials of novel glioma therapies (18).

**Figure 1:**
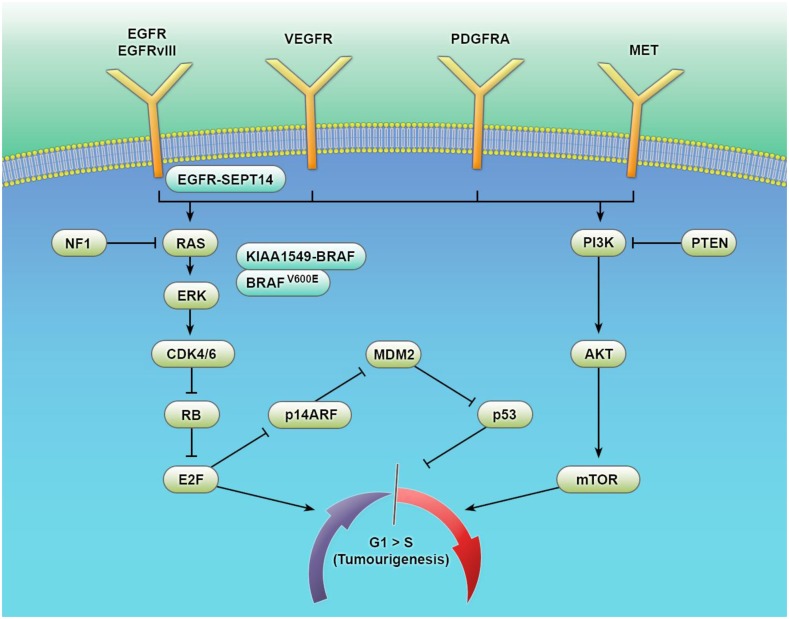
**Key signaling pathways in the tumorigenesis of gliomas**. The biological behavior of glial tumors is traditionally based on histological typing and grading. Histology is increasingly being supplemented using molecular information on genes, epigenetic markers, transcriptional regulators, and complex gene signatures. This multitude of events converges on a number of common signaling pathways with considerable cross talk. Increased understanding of these pathways and their interactions is facilitating a biomarker-driven approach to glial tumor biology – improving diagnosis, prognostic estimation, and the development of targeted therapies.

In this review (see Figure 1 and Table 1), we will consider recent advances in our understanding of adult and pediatric gliomas, highlighting both the prospectively validated prognostic and predictive biomarkers (other than tumor grade and morphology) in current use. Additionally, putative biomarkers will be discussed with relevance to both future clinical utility and our current understanding of gliomagenesis.

**Table 1:** **Tissue biomarkers with current clinical or promising relevance in malignant glioma**.

Biomarker	Diagnostic	Prognostic	Predictive	Comment
**ADULT GLIOMAS**				
IDH1 mutations	+	+	−	DNA sequence or protein level biomarker; commonly mutated in low grade and secondary GBM (20); negative in non-neoplastic glioma mimickers; has prognostic value in WHO Grade III and IV GBM (22, 23)
MGMT promoter methylation	(+)	+	+	An epigenetic biomarker in GBM with a putative diagnostic role in detecting pseudoprogression (190); indicates an improved prognosis in malignant glioma (191) and predicts an improved PFS with TMZ chemotherapy and RT vs. RT alone (52, 53)
1p/19q Co-deletion	+	+	+	Chromosome level biomarker commonly found in oligodendroglial tumors (192); associated with an overall better prognosis (193) and predicts improved survival benefit in response to chemotherapy and RT vs. RT alone (68, 69)
EGFR amplification/EGFRvIII mutation	(+)	(+)	(+)	A gene level biomarker frequently altered in GBM (194); with putative prognostic validity (103–105); a potential predictive biomarker for molecular therapies (113)
PTEN	−	+	(+)	A gene level biomarker, LOH of which is associated with poor survival outcomes for both anaplastic astrocytomas and GBM (92). The lost or inactive state of PTEN has been linked to the resistance of targeted EGFR inhibitors in GBM (93)
PDGFRA	−	+	−	A gene level biomarker; PDGFRA amplification has recently been shown to be associated with a poor prognosis in IDH1 mutant GBM (117) and have a negative prognostic value in Grade III gliomas (118)
**PEDIATRIC GLIOMAS**				
KIAA1549:BRAF	+	+	−	A gene level biomarker commonly detected in PA (153) and PMA (167); has prognostic validity in PA and WHO Grade II astrocytomas (156)
BRAF^V600E^	+	(+)	(+)	A gene level biomarker commonly seen in supratentorial PA, PXA, or GG (154); has putative roles as a prognostic (165) and predictive biomarker (166). Vemurafenib may improve outcomes in adults with recurrent BRAF^V600E^ mutated PXA (170)
K27M-H3.3	−	+	−	An epigenetic biomarker conferring a worse OS in DIPG (145)

*^+^Current evidence based glioma biomarker*.

*^(+)^Putative glioma biomarker under current investigation*.

*TMZ, temozolomide; RT, radiotherapy; PFS, progression free survival; OS, overall survival; GBM, glioblastoma multiforme; AO, anaplastic olidogendroglioma; PA, pilocytic astrocytoma; PMA, pilomyxoid astrocytoma; PXA, pleomorphic xanthoastrocytoma; GG, ganglioglioma; DIPG, diffuse intrinsic pontine glioma*.

## Biomarkers in Adult Gliomas

### Primary vs. secondary gliomas and isocitrate dehydrogenase (IDH1 and IDH2)

Isocitrate dehydrogenases are members of a group of enzymes involved in energy metabolism that catalyze the decarboxylation of isocitrate (ICT) into α-ketoglutarate (19). Several studies have confirmed that IDH1 is mutated in approximately 60–80% of diffusely infiltrating gliomas (WHO Grade II and III) and in secondary GBMs, which may derive from them (20). However <10% of primary GBM carry this mutation (20, 21).

Although patients with IDH1 mutations are generally younger, a multivariate analysis has confirmed that IDH1 mutation can be considered a favorable independent prognostic marker in Grade III and IV gliomas, but not in WHO Grade II astrocytomas (22, 23). IDH2 (isoenzyme) mutations are less common than IDH1 mutations and confer a similar improved prognosis, but as yet the precise mechanism for this has not been elucidated (20, 24).

Watanabe et al. reported that IDH1 mutations always precede TP53 mutations or 1p/19q loss (see below) in a large cohort of Grade II/III and secondary gliomas (25). Disruption of the p53 pathway disruption is frequent in gliomagenesis [dysregulated in 85.3% of GBM according to the latest TCGA findings (15)] and TP53 mutations play a particularly important role in the development of secondary GBM (26). The prognostic significance of mutated TP53 in GBM is unclear (27, 28). However, it has been shown that mutant TP53 is associated with increased activity of a homologous recombination-mediated mechanism called alternative lengthening of telomeres (ALT). This confers a more benign biology and favorable prognosis (29, 30).

The ALT phenotype has been positively correlated with IDH mutations, TP53 mutations, and mutations of the alpha thalassemia/mental retardation syndrome X-linked (ATRX) gene in astrocytomas but not in the oligodendroglial tumor lineage (31, 32). ATRX encodes a subunit of the chromatin remodeling complex, which is key for H3.3 incorporation into heterochromatin at centromeres and telomeres (33).

In oligodendrogliomas, a high frequency of TERT promotor mutations have been reported (34), occurring concurrently with IDH mutations and total 1p/19q loss [resulting in the loss of the tumor suppressor genes CIC and far upstream element binding protein 1 (FUBP1) – see below] (35). TERT is a catalytic subunit of telomerase and occurs mutually exclusively with ATRX mutations (34). This suggests that ATRX and TERT promotor mutations serve as alternative mechanisms for telomere lengthening.

This recent evidence points to two recurrent genetic signatures in gliomas. The first group progresses along an astrocytic lineage with ALT, alterations in ATRX, TP53, and IDH. The second group has a strong oligodendroglial component and carries IDH mutations and alterations in either CIC and FUBP1 and/or 1p/19q loss. The latter group has the longest median overall survival (OS) (36).

These finding support current theories that IDH1 mutation occurs early in gliomagenesis and may affect a glial proneural cell population that can give rise to both astrocytes (with ATRX and TP53 mutations) and oligodendrocytes (with TERT mutations and 1p/19q co-deletions) (37). Most recently, TERT mutations have been shown to have a significant overlap with IDH1 wild type primary GBM (38), suggesting that alternative progenitor cells give rise to these tumors.

A remarkable study showed that the R132H IDH1 point mutation results in the production of a different functional metabolite called 2-hydroxyglutarate (2-HG) (39). Increased 2-HG is known to inhibit histone demethylation (40), in part because histone demethylases and TET 5-methylcytosine hydroxylases are α-ketoglutarate-dependent dioxygenases involved in epigenetic control (41). Mutated IDH1 and increased 2-HG may therefore pre-dispose glioma cells to DNA hypermethylation.

Extensive DNA hypermethylation can be part of the so-called glioma CpG island methylator phenotype (G-CIMP) (42). A recent study reported a significant association between IDH mutation and methylation status (determined by the H3k9me3 methylation mark) in Grade II but not Grade III (anaplastic) astrocytomas or glioblastomas (43). Furthermore, H3K9me3-positive Grade II oligodendrogliomas showed improved OS when compared with H3K9me3-negative cases (43).

Glioma CpG island methylator phenotype GBM has been shown to be associated with younger patients, with IDH1 mutations and an improved prognosis, clustering in the TCGA proneural subgroup (44). The G-CIMP phenotype therefore appears to be a feature of lower grade gliomas and provides a molecular definition of secondary glioblastoma (45). It may be possible in the future to monitor mutant IDH1 glioma-associated 2-HG using non-invasive MR spectroscopy (46). Furthermore, the early role of mutant IDH in neoplastic transformation has lead to development of mutant IDH specific inhibitors (47). IDH could therefore assume an additional role as a predictive biomarker.

### Epigenetic modifiers of glioblastoma – MGMT promoter methylation and miRNAs

Recently, epigenetic modifiers, such as hypermethylation have been implicated in the significant differences in glioma response to treatment (48). Of particular importance in glioblastoma is the DNA repair protein *O*^6^-methlyguanine methyl transferase – MGMT – (10q26). Active MGMT is able to remove alkyl groups from DNA, thereby reducing the efficacy and promoting cancer cell resistance to alkylating chemotherapeutic agents such as temozolomide (TMZ) (49).

Following on from the work of Esteller et al. (50), a landmark paper by Hegi et al. showed that MGMT promoter methylation silences gene expression in 45% cases of GBM. (51). This results in a significantly better prognosis for patients.

Following this discovery, the EORTC-NCIC trial and other studies have shown that MGMT promoter methylation increases progression free survival (PFS) in cases of GBM treated with TMZ and radiotherapy (RT) vs. RT alone (52, 53). Variability in methylation load at individual CpG sites also seems to affect PFS and OS in patients receiving TMZ (54).

In addition, the prospective randomized NOA-08 (55) and Nordic (56) trials showed that MGMT promoter methylation in elderly patients (who traditionally receive RT alone for malignant astrocytoma) correlates with longer event free survival and OS (respectively) when treated with TMZ. The latest TCGA findings suggest that MGMT promoter methylation may only have such predictive validity in classical subtype GBM (15). A multivariable analysis has additionally shown that the MGMT promoter methylation (MGMT-STP27) status and G-CIMP phenotype have a significant prognostic role in anaplastic oligodendrogliomas/oligoastrocytomas and are predictive of OS outcomes when treated with adjuvant procarbazine/CCNU/vincristine (PCV) (see below) chemotherapy (57).

Further epigenetic regulation is imposed on gliomas by miRNAs exerting complex effects on cancer gene networks (12). miRNAs may exert tumor suppressive or oncogenic functions through the post-transcriptional regulation of gene expression. miR-21 was the first miRNA to be investigated in gliomas (58) and is elevated in gliomas compared to healthy cortex (59). miR-21 acts as an oncogenic miRNA, inhibiting matrix metalloproteinase regulators and promoting glioma cell migration (60). miR-181a/b/c were originally found to be downregulated in glioblastoma (61). Of note, downregulation of miR-181b and miR-181c has been associated with clinical response to RT and TMZ when compared to patients with progressive disease (62). miR-181d has been shown to exert a suppressive effect on MGMT expression with a corresponding inverse association with TMZ response in GBM (63). Most recently, this has been reported to occur due to the effect of miR-181d on MGMT protein translation, downregulating MGMT expression independently of promoter methylation (64). miRNA data has also identified GBM subclasses within the TCGA, with significant clinical differences (65). Interestingly, miRNAs (such as miR-9) were shown to regulate subclass-specific gene expression and thus characterize and contribute to the phenotypic diversity of glioblastoma subclasses. Full discussion of miRNAs implicated in glioma initiation and progression are out with the scope of this review. Nevertheless, miRNAs present exciting opportunities for further biomarker research with direct application for patient stratification in clinical trials or as future therapeutic agents (58).

### Co-deletion of chromosomes 1p/19q in oligodendrogliomas and gross chromosomal alterations in GBM

Loss of the short arm of chromosome 1 and the long arm of chromosome 19 (co-deletion of 1p/19q) is seen in >60% of Grade II/III oligodendroglial tumors (66, 67).

The recent findings of the major randomized clinical trial RTOG 9402 showed an OS benefit in response to PCV chemotherapy plus RT vs. RT alone in patients with 1p/19q co-deletions and both anaplastic oligodendroglioma and anaplastic oligoastrocytoma (68). A recent update of the similar EORTC 26951 trial confirmed that patients with 1p/19q co-deleted anaplastic oligodendroglial tumors showed an improved OS and PFS when treated with adjuvant PCV (69).

The cause for this survival benefit is still unclear. However, recently two candidate tumor suppressor genes have recently been discovered within the areas of chromosomal loss. FUBP1 and CIC (the homolog of the *Drosophila capicua* gene) have been identified on 1p and 19q, respectively (70, 71). FUBP1 is thought to be a dual (activator/inhibitor) transcriptional modulator of MYC (72). CIC is thought to act as a transcriptional repressor, regulating target gene expression of activated epidermal growth factor receptor (EGFR), Ras/Raf, and MAPK cancer pathways (73).

In previous studies, the combination of 1p and 19q loss in GBM is rare, and numbers are too small to reliably determine any survival benefit (28). However, loss of 1p alone has been observed in both primary and secondary GBM (74). Interestingly, a multivariate analysis found that LOH 1p alone was associated with longer GBM survival (75). Detailed studies have shown that 1p36 is a preferential target of chromosome 1 deletions in astrocytic tumors and homozygously deleted in a subset of glioblastomas (76). It has been also been suggested that 19q LOH alone may confer a longer OS in patients with GBM (77).

The short arm of chromosome 9 contains the tumor suppressor genes CDKN2A and CDKN2B, which encode p14^ARF^, p16^INK4A^, and p15^INK4B^. These proteins have key roles in the p53 and RB pathways and hence, the G1 cell cycle checkpoint. LOH 9p is frequently seen in GBM samples (78, 79) and has been associated with a shorter OS (77) although other studies have not confirmed this finding (80). Interestingly, the absence of CDKN2A or CDKN2B deletion and a frequent loss of 17p (which includes p53) differentiate diffuse intrinsic pontine glioma from non-brainstem high-grade pediatric glioma (see later) (81).

Most recently, a small subset of GBMs has been reported as carrying chromosomal translocations fusing the tyrosine kinase domains of FGFR genes (FGFR1 or 3) to TACC1 or 3, respectively (82). The resulting fusion protein localizes to metaphase spindle poles, inducing aneuploidy. An FGFR inhibitor (83) was shown to prolong survival in FGFR3-TACC3-initiated glioma mice models. This gives promise to FGFR-TACC rearrangements as putative predictive biomarkers for FGFR inhibitor response (84).

### EGFR activation, EGFRvIII mutation, and downstream mediators of GBM tumorigenesis

It is well known up to 65% of so-called primary glioblastomas show epidermal growth factor receptor (7p12) amplification, overexpression, and/or mutations of this pathway (10). Such glioblastomas are part of the “classic” expression subtype (10) occurring mutually exclusively with IDH-mutated secondary GBM (20). EGFR is a receptor tyrosine kinase. Ligand binding by EGF promotes receptor dimerization and autophosphorylation of the cytoplasmic domain (85). Such EGFR activation is thought to promote cellular proliferation via activation of the MAPK and PI3K-Akt pathways (86).

The small molecule inhibitors gefitinib and erlotinib compete with ATP to prevent phosphorylation and therefore, ameliorate oncogenic downstream signaling (87). However, this mitogenic signaling network shows significant modularity in GBM (88). Gliomas are therefore able to escape the need for receptor function by activating alternative oncogenic pathways when challenged by receptor targeted-agents (89), dramatically revealing the complexity of tyrosine kinase signaling in these tumors (85). This means that treatments targeted to multiple signaling hubs and inhibitors of compensatory signaling paths are needed to inhibit tumor growth and recurrence.

Previous trials of EGFR kinase inhibitor therapy have been unsuccessful (90), most likely due to the role of downstream mediators of the pathway, which can undergo mutation (91). For example, PTEN is a tumor suppressor gene located at 10q23. It functions to suppress the PI3K-Akt signaling pathway. LOH at 10q is associated with poor survival outcomes for both anaplastic astrocytomas and GBM (92) and the lost or inactive state of PTEN has been linked to the resistance of targeted EGFR inhibitors in GBM (93). However, *in vitro* studies have shown that PTEN deficient GBM cells show significant responses to combined mTOR/EGFR kinase inhibitor therapy (94, 95).

Further predictive response stratification may be possible through baseline measurements of downstream mTOR activators such as s6 kinase (96). Similarly in GBM patients, levels of phosphorylated Akt have been shown to be predictive of clinical response to erlotinib in EGFR amplified tumors (97, 98). This highlights the importance of carefully selected molecular determinants for rational trials of EGFR inhibitors in GBM. Downstream pathway inhibitors such as BMK120 – a pan PI3K inhibitor currently undergoing phase II trials in patients stratified by PTEN status (99) – may also have efficacy when used in combination with EGFR inhibitors. Furthermore, gain-of-function mutations in PI3KCA have been found in 15% of glioma samples (100) and pre-clinical studies have shown that dual PI3KCA/mTOR inhibitors augment the antiproliferative effects of EGFR inhibition (101). Such combination therapy could be used in concert with traditional chemo- and radiotherapy to abrogate alternative survival pathway activation and enhance cytotoxicity (102).

The evidence for EGFR amplification as an independent predictor for survival in GBM varies between studies (103–105). However, higher expression of the down stream mediator phosphorylated MAPK has been reported to independently confirm a lower OS in newly diagnosed GBM (106).

Of current interest is the EGFRvIII mutation, which is the most common EGFR mutation resulting from an in-frame deletion of 801bp spanning exons 2–7 (107). This leads to a constitutively active EGFR (107). This EGFRvIII mutation occurs in 20–30% of GBM patients and is detected exclusively in cases with EGFR amplification (108).

Interestingly, *in vitro* models have suggested that GBM cells expressing EGFRvIII are resistant to small molecule tyrosine kinase inhibitors (109). EGFRvIII signals to a mTOR complex 2 induced mechanism (110), thereby differing from the EGFR-mTOR complex 1 signaling axis, which may contribute to such therapy resistance. Nevertheless, co-expression of EGFRvIII and wild type PTEN has previously been shown to be associated with response to erlotinib in GBM patients (111). Conversely, erlotinib has been suggested to act synergistically with a c-Met inhibitor against *in vitro* models lacking PTEN (112). An ongoing trial of the EGFRvIII vaccine (Rindopepimut) CDX-110 has shown longer OS in patients treated after GBM resection (113).

Recently, an analysis of in-frame gene fusions in GBM has identified EGFR-SEPT14 fusions, which tend to cluster in the TCGA classical and mesenchymal subgroups, constitutively activate STAT3 signaling and confer mitogen-independent growth (114). EGFR-SEPT14-positive GBM xenograft cells additionally showed a significant response to lapatinib (114), indicating a predictive role for EGFR fusions in EGFR inhibitor clinical trials. The latest whole genome sequencing work from the TCGA has identified further complex rearrangements of the EGFR gene or structural variants of genes surrounding its locus (15). The prognostic and predictive effects of these rearrangements have yet to be elucidated.

In GBM, platelet-derived growth factor receptor alpha (PDGFRA) is the most commonly altered receptor tyrosine kinase after EGFR (10, 115). GBM with amplified PDGFRA have been shown to be associated with either amplified EGFR or amplified MET (the hepatocyte growth factor receptor) (116). PDGFRA amplification has recently been shown to be associated with a poor prognosis in IDH1 mutant GBM (117) and have a negative prognostic value in Grade III gliomas (118). In PDGFRA amplified gliomas two genetic rearrangements have been described – a gene fusion between kinase insert domain receptor (KDR) and the PDGRFA gene and PDGFRA (Δ8, 9), an intragenic deletion rearrangement (115). Both PDGFRA mutants behave as transforming oncogenes with elevated tyrosine kinase activity. Unfortunately, early phase clinical trials of dual tyrosine kinase and PDGFR inhibitors have not been successful (119), indicating that a better understanding of this signaling pathway is needed prior to further clinical testing.

### Vascular endothelial growth factor and its receptors

Vascular endothelial growth factor (VEGF) plays a key role in the malignant angiogenesis seen in GBM. Activated vascular endothelial growth factor and its receptors 2 (VEGFR2) activates the Ras-Raf and PI3K-Akt transduction pathway (120). Although >60% of GBM express VEGF by immunohistochemistry, VEGF expression does not correlate with survival in GBM (121). However, shorter PFS has been associated with increased VEGFR2 immunohistochemical expression at tumor recurrence (122).

Bevacizumab (Avastin) is a humanized monoclonal antibody against VEGF (123). The RTOG-0825 (US) and AVAGlio (Europe) trials are large randomized phase III trials using bevacizumab in addition to standard radio- and chemotherapy in newly diagnosed GBM (124). These trials showed no improvement in OS with bevacizumab (125, 126). However, subgroup and molecular analysis may still reveal positive results (127) as could prospective randomized trials of combination therapies that include bevacizumab (128).

The tyrosine kinase inhibitor sorafenib is currently being trialed in combination with mTOR inhibitors in patients with recurrent GBM (129) and with standard chemotherapy and RT in untreated GBM (130). Sorafenib has multi-target activity against VEGFR2, VEGFR3, PDGFRB, c-Kit, BRAF, and Raf (131). The clinical response outcomes have so far been disappointing in recurrent GBM (132). Sunitinib is an alternative multi-target inhibitor against VEGFR1-3, PDGFRA/B, FLT-3, c-Kit, and RET (133, 134). Unfortunately, results so far indicate no significant improvement in PFS in recurrent GBM (135).

## Biomarkers in Pediatric Gliomas

### Pontine, hemispheric, and thalamic gliomas

Although pediatric GBM are morphologically indistinguishable from adult GBM, they have a unique biological signature (136). For example, while TP53 and PIK3CA mutations occur in all high grade gliomas (137), <10% of childhood GBM harbor EGFR amplifications or PTEN mutations (138, 139). Similarly, IDH mutations are found in <5% of pediatric GBM although become more common in adolescents (140).

K27M and G34V/R gain-of-function mutations in H3F3A, which encodes histone H3.3, have recently been studied in pediatric and young adult GBM (141, 142). H3.3 is known to modulate gene expression during brain development (143). H3.3 mutations were shown to be prevalent in pediatric cohorts and mutually exclusive with IDH mutations (142, 144). K27M mutations have been shown to occur in over 70% of pediatric diffuse intrinsic pontine gliomas (DIPG) and confer a worse OS when compared to patients with wild type H3.3 (145). Additionally, 80% of pediatric thalamic GBM carry this mutation (145). Indeed, K27M-H3.3 mutations characterize pediatric midline GBM (136). In cohorts of childhood brainstem and thalamic tumors, this mutation occurs commonly in the younger (median 10 years) age group (142, 145), overlaps significantly with TP53 but only co-occurs with ATRX mutations in approximately half of all cases (145). The K27M mutation has also been found in the related histone H3.1 in 18% of DIPG (141).

G34V/R H3.3 mutations have been noted to cluster in childhood hemispheric GBM (144, 145). Such supratentorial tumors occur in an older population (median 18 years) and almost always overlap with TP53 and ATRX mutations (142). Recently, it has been reported that loss-of-function mutations in the H3K36 trimethyltransferase SETD2 also occur in supratentorial high grade gliomas of older children and young adults and are mutually exclusive with H3.3 mutations (146).

How H3.3 mutations promote tumorigenesis through interference with chromatin function has not been fully elucidated. However, the K27M mutation has been shown to inhibit K27 methylation (associated with polycomb-mediated gene repression) (147) and K27 acetylation (which is present on active gene promoters) (148). Both H3.3 mutations may additionally alter the expression of neural development genes (149). Furthermore, future work on the role of H3K36 methylation in tumorigenesis and the effects of differing histone mutations may reveal roles as predictive biomarkers (146).

### Pilocytic astrocytomas: KIAA1549:BRAF and BRAF^V600E^

Pilocytic astrocytomas are the most common childhood brain tumor, accounting for approximately 20% of brain tumors under the age of 20 (150). Pilocytic astrocytomas are slow-growing, non-infiltrating tumors with WHO malignancy Grade I (2). They may occur throughout the central nervous system but are found most frequently in the posterior fossa and the hypothalamic/chiasmatic region. Gross surgical resection frequently results in a cure and over 90% of patients survive more than 10 years (151). However, local recurrence occurs in 10–20% of cases and the primary lesion, its recurrence and the subsequent treatment can cause considerable morbidity as well as neurocognitive and endocrine dysfunction (152).

Jones et al. (153) were the first to describe a tandem duplication at 7q34 in a high proportion of pilocytic astrocytomas. This rearrangement creates a fusion gene (KIAA1549:BRAF) with constitutive BRAF kinase activity and putative abnormal activation of MAPK/ERK pathway. KIAA1549 is an as yet uncharacterized gene.

Multiple exonic fusion combinations between KIAA1549 and BRAF have now been reported, all of which result in loss of the BRAF autoregulatory N-terminal domain while the C-terminal kinase domain is retained, resulting in constitutive activation of the oncogenic MAPK pathway (154). The frequency of this fusion gene in pilocytic astrocytomas reported in the literature varies from 50 to 100%, depending on the patient demographic (154). The genetic mechanism resulting in this fusion is the subject of ongoing research (155).

It was originally reported that there was no significant difference in survival at follow up of fusion positive vs. fusion negative pilocytic astrocytomas (153). Hawkins et al. later found that the KIAA1549:BRAF fusion was an independent prognostic marker for significantly improved 5 year PFS for pilocytic astrocytomas as well as Grade II diffuse and pilomyxoid astrocytomas (156).

The KIAA1549:BRAF fusion gene was initially thought to be highly specific for pilocytic astrocytomas (154) thereby suggesting a useful diagnostic biomarker for a tumor with varied and challenging histology. However, Lin et al. recently identified KIAA1549:BRAF in 36% of glioneuronal tumors studied as well as 33% of pilomyxoid astrocytomas (157). In addition, KIAA1549:BRAF has been identified in 9% of diffuse adult gliomas (pre-dominantly oligodendroglial neoplasms) (158). In a large proportion of these cases, KIAA1549:BRAF and IDH mutations were present as independent molecular events. This suggests that in a small proportion of adult glial tumors 7q34 rearrangements may contribute to Ras-RAF-ERK signaling dysregulation. This has important implications for the design of novel therapies.

The above discussion highlights the single pathway nature of pilocytic astrocytomas – a disease driven by abnormal MAPK/ERK pathway activation. Indeed, recent evidence has highlighted further oncogenic “hits” driving this pathway. Two new fusion genes involving the kinase domain of the known oncogene NTRK2 and point mutations within the kinase domain of FGFR1 have been identified (159). All FGFR1-mutant tumors are extracerebellar and may be further modified by co-expression of the mutated phosphatase gene PTPN11 to upregulate phosphorylated ERK (159). NTRK2, FGFR1, and PTPN11 are likely to act as upstream drivers of MAPK/ERK pathway activation and may be responsible for the paradoxical pathway activation seen in KIAA1549:BRAF tumors treated with BRAF inhibitors (160). These genetic changes could therefore become key as predictive biomarkers for stratifying patients in ongoing trials of combination FGFR, NTRK2, and/or MAPK/ERK kinase inhibitors (161, 162).

Infratentorial posterior fossa pilocytic astrocytomas tend to display a high frequency of the KIAA1549:BRAF fusion (163). KIAA1549:BRAF fusion positive pilocytic astrocytomas also occur less frequently with increasing age (164). Supratentorial tumors are less frequently fusion positive but have an increased frequency of the oncogenic BRAF^V600E^ mutation (154), a putative pediatric low grade prognostic biomarker (165), and promising predictive biomarker (166).

Additionally, BRAF ^V600E^ is associated with 60–80% of pleomorphic xanthoastrocytomas (PXA, WHO Grade II) across all age groups (167). These tumors do not have concomitant 7q34 BRAF duplications (168), indicating a potential diagnostic role for the BRAF^V600E^ mutation. A BRAF^V600E^ mutation specific antibody is available (169) and a recent small study suggested that vemurafenib (a BRAF inhibitor with significant activity against BRAF mutated metastatic melanoma) may improve outcomes in adults with recurrent BRAF^V600E^ mutated PXA (170). BRAF^V600E^ is also reported in 20–25% of pediatric and adult gangliogliomas (171) [or higher (172)] and may have a role as a negative prognostic biomarker (173). BRAF^V600E^ mutated gangliogliomas have been shown to be associated with concomitant mTOR pathway activation, which may aid the development of targeted treatments for this tumor (174).

### Gliomas, intratumoural heterogeneity, and brain tumor stem cells

The discussion above highlights our developing understanding of glial tumors as entities with individual pathological molecular events and treatment responses. It is increasingly recognized, however, that individual tumors contain distinct spatial regions with differing molecular profiles. For example, microdissected glioma tissue specimens have been shown to harbor unique molecular profiles in central vs. peripheral invasive regions (175). Receptor kinases (EGFR, MET, PDGFRA) have also been found to be amplified in single tumors in different cells in a mutually exclusive fashion (176). Such intratumoural heterogeneity results in divergent subclones of neoplastic cells within the same tumor and is likely to drive treatment failure, treatment resistance, and subsequent recurrence of the malignancy (177, 178). Indeed, it has recently been shown that different samples from the same GBM can be classified into different GBM subtypes (179). It follows that the impact of sampling bias must be considered when stratifying patients in future clinical trials using molecular criteria.

Furthermore, tumor recurrence post-treatment may consist of multiple neoplastic clones coexisting in the same lesion. Analysis of such patterns of heterogeneity could enable patients to receive targeted multimodal therapies for recurrent tumors (12, 180). This aim will additionally require a greater understanding of how brain cancer stem cells (also termed brain tumor initiating cells) drive such divergent neoplastic clones and cause tumor relapse (181–183). It has been shown both *in vivo* and *in vitro* that brain tumor initiating cells can be epigenetically differentiated into mature neuronal type cells (184, 185). Such cells can also be directed into non-neuronal cell types with a resultant suppression of malignant cellular behavior (186). It is hoped that such epigenetic manipulation could 1 day lead to further, more refined, personalized treatment (187, 188).

## Conclusion

Over the last decade, there has been an unprecedented surge in our understanding of what drives neoplastic growth in glial tumors. Further molecular characterization of these tumors in the future will accelerate biomarker discovery and facilitate the creation of new diagnostic categories for gliomas (189). Only IDH mutation status (prognostic) and MGMT methylation status and 1p/19q co-deletion (predictive) are currently routinely used for evaluation of glioma patients by clinicians in the US and UK. However, the ongoing development of targeted therapies as mono and combination treatments necessitates the discovery of optimal molecular predictive biomarkers, which will further our understanding of these tumors.

Additionally, biomarker analysis will become a major factor in glioma clinical trials, with rapid identification of putative biomarkers in early stage trials with sufficient statistical design to validate predictive associations in phase III trials. Care will therefore be required to distinguish biomarkers that provide prognostic information from those that have predictive validity. This approach will allow us to determine future personalized therapeutic choices with minimal toxicity and improve clinical outcomes for patients for whom the diagnosis of a malignant glioma still portends a dismal outlook.

## Author Contributions

Harry R. Haynes: manuscript research and writing; Sandra Camelo-Piragua: manuscript review and revision; Kathreena M. Kurian: manuscript research and writing.

## Conflict of Interest Statement

The authors declare that the research was conducted in the absence of any commercial or financial relationships that could be construed as a potential conflict of interest.
